# Early structural changes of the heart after experimental polytrauma and hemorrhagic shock

**DOI:** 10.1371/journal.pone.0187327

**Published:** 2017-10-30

**Authors:** Christian K. Braun, Miriam Kalbitz, Rebecca Halbgebauer, Philipp Eisele, David A. C. Messerer, Sebastian Weckbach, Anke Schultze, Sonja Braumüller, Florian Gebhard, Markus S. Huber-Lang

**Affiliations:** 1 Institute of Clinical and Experimental Trauma-Immunology, University Hospital of Ulm, Ulm, Germany; 2 Department of Orthopedic Trauma, Hand-, Plastic- and Reconstructive Surgery, University Hospital of Ulm, Ulm, Germany; 3 Department of Orthopedic Surgery, University Hospital of Ulm, Ulm, Germany; University of PECS Medical School, HUNGARY

## Abstract

Evidence is emerging that systemic inflammation after trauma drives structural and functional impairment of cardiomyocytes and leads to cardiac dysfunction, thus worsening the outcome of polytrauma patients. This study investigates the structural and molecular changes in heart tissue 4 h after multiple injuries with additional hemorrhagic shock using a clinically relevant rodent model of polytrauma. We determined mediators of systemic inflammation (keratinocyte chemoattractant, macrophage chemotactic protein 1), activated complement component C3a and cardiac troponin I in plasma and assessed histological specimen of the mouse heart via standard histomorphology and immunohistochemistry for cellular and subcellular damage and ongoing apoptosis. Further we investigated spatial and quantitative changes of connexin 43 by immunohistochemistry and western blotting. Our results show significantly increased plasma levels of both keratinocyte chemoattractant and cardiac troponin I 4 h after polytrauma and 2 h after induction of hypovolemia. Although we could not detect any morphological changes, immunohistochemical evaluation showed increased level of tissue high-mobility group box 1, which is both a damage-associated molecule and actively released as a danger response signal. Additionally, there was marked lateralization of the cardiac gap-junction protein connexin 43 following combined polytrauma and hemorrhagic shock. These results demonstrate a molecular manifestation of remote injury of cardiac muscle cells in the early phase after polytrauma and hemorrhagic shock with marked disruption of the cardiac gap junction. This disruption of an important component of the electrical conduction system of the heart may lead to arrhythmia and consequently to cardiac dysfunction.

## Introduction

Severe traumatic injury is the leading cause of death in adolescence globally [1] and the total number is likely to increase until the year 2020 [2]. Often, these injuries cause traumatic bleeding, leading to early hypovolemia and shock, thus presenting a severe condition worsening the outcome for polytrauma (PT) patients [3].

The concept of a wide-spread activation of the immune system immediately after severe tissue trauma has long been established with ongoing research on the resulting activation of various protease cascade systems in the blood, which aggravates the patient’s condition [4]. A similar systemic inflammatory response occurs during sepsis, which can trigger a secondary cardiomyopathy. The underlying pathomechanisms include interactions of cytokines with their respective receptors on cardiomyocytes, and possibly by effects of locally and systemically acting complement components [5,6]. However, the concept of an early development of trauma-driven cardiomyopathy, resulting from the posttraumatic systemic inflammatory response remains to be proven and examined in detail. In a recent clinical study, post-injury cardiomyopathy occurred in 20% of the fatal cases after severe trauma and histopathological examination of heart specimens revealed significant myocardial lesions even in the absence of coronary alterations [7]. Most likely, these changes resulted from the patient’s reaction to ischemia and shock conditions without a direct injurious impact on the heart [7].

A high serum level of cardiac troponin I (cTnI) as a specific marker of cardiac damage has recently been demonstrated as an independent risk factor for the outcome of patients, admitted after severe trauma [8]. Furthermore, high-mobility group box 1 (HMGB1), which functions as a damage-associated molecular pattern (DAMP) and is actively secreted as a danger response signal by a variety of cells [9], has recently been proposed to contribute to inflammation-mediated cardiac dysfunction via toll-like receptor 4 interaction [10]. Cardiac gap junctions (CGJ), which mainly consist of connexin 43 (Cx43) polymers are essential in electric coupling of the myocardium and for synchronized heart activity [11]. Recent studies in septic patients found disruption of the CGJ with lateralization of Cx43 across the plasma membrane [12] which could result in impaired electrophysiological coupling. However, it remains elusive whether these changes also appear in the early phase after PT with an additional severe hemorrhagic shock (HS).

In the present study, we applied a clinically relevant rodent model of PT and HS [13] to investigate the impact of early post-traumatic inflammation on the molecular and structural integrity of the heart.

## Material and methods

### Animals and model of polytrauma and hemorrhagic shock

In the interest of limiting animal numbers, we took the heart samples from a recently conducted prospective randomized experimental study, published by our group [13], including the detailed evaluation of the study protocol. Here, we assessed morphological and functional changes in the heart which were not in the focus of the main study. The study protocol was approved by the University Animal Care Committee and the Federal Authorities for animal research, Tübingen, Germany (No. 1194) and performed following the National Institutes of Health Guidelines for the use of laboratory animals.

In brief, randomly assigned male C57BL/6 mice (Jackson Laboratories, USA) were subjected to PT, consisting of airblast-induced blunt chest-trauma, traumatic brain injury and closed femoral fracture and subsequent insertion of femoral artery and jugular vein catheters and pressure-controlled HS. Mice were anaesthetized with 2.5% sevoflurane (Sevorane Abbott, Germany) and 97.5% oxygen throughout the procedure and were given 0.03 mg/kg buprenorphine by subcutaneous injection for analgesia. After induction of experimental trauma, the animals were bled to reach a mean arterial blood pressure (MAP) of 30 mmHg (± 5 mmHg) which was kept stable for 60 min. Subsequently, volume resuscitation was performed by infusion with the fourfold of the drawn blood-volume with Ionosteril (Fresenius-Kabi, Germany) over 30 min and were further observed for 2 h. A goal MAP of 50 mmHg or higher was maintained by additional volume infusion and administration of norepinephrine (Sanofi, Germany) as required. For euthanization, animals were given 5% sevoflurane, were bled out and received a bilateral pneumothorax. Randomly selected healthy animals served as native controls (CTRL).

### Enzyme-linked immunosorbent assay (ELISA) analyses and blood-gas analysis

To assess plasma concentrations of cTnI, keratinocyte chemoattractant (KC) and monocyte chemotactic protein-1 (MCP-1), sandwich-enzyme-linked immunosorbent assay (ELISA) based kits were used adhering to manufacturers’ instructions: mouse CXCL1/KC DuoSet ELISA Kit (R&D Systems, Germany), Ultra-Sensitive Mouse Cardiac Troponine-I ELISA (Life Diagnostics, USA), Mouse MCP-1 ELISA Kit (BD Biosciences, USA). For determining plasma C3a levels, a monoclonal rat anti-mouse anti-C3a antibody (BD Biosciences, USA) was used for antigen detection according to standard ELISA protocol. Recombinant mouse complement component C3a (R&D Systems, Germany) served for calculation of a standard curve.

To rule out dilution effects due to resuscitation procedure, all results were normalized to the respective amount of total plasma protein. Total amount of protein in plasma was evaluated, using a Pierce Bicinchoninic Acid (BCA) Assay (Thermo Fisher Scientific, USA).

Blood hemoglobin was evaluated by standard blood gas analysis (ABL 700 blood gas analysis device; Radiometer, Denmark).

### Western blotting

Left ventricle specimens were thawed, homogenized and diluted with proteinase inhibitor and radioimmunoprecipitation assay lysis buffer (all reagents purchased from Sigma-Aldrich, USA) prior to further processing. To assess the amount of total protein per specimen, a Pierce BCA assay was performed (Thermo Fisher Scientific, USA). Homogenates were diluted in sample buffer to yield 30 μg protein per sample, heated for 7 min and loaded onto gels. Electrophoresis was performed at 200 V for 40 min and gels were afterwards blotted to nitrocellulose membranes (Bio-Rad, USA). Membranes were blocked with 5% milk for 90 min at room temperature and incubated with a polyclonal rabbit anti-Cx43 antibody (Cell Signaling Technologies, USA) over night at 4°C.

Afterwards, membranes were washed with 0.1% tween in tris-buffered saline and antibody detection was performed with horseradish peroxidase linked goat anti-rabbit secondary antibody and StrepTactin 0.4% (both Bio-Rad, USA) and developed using a Clarity Western electrochemiluminescence solution (Bio-Rad, USA) prior to imaging.

Bands were detected using the ChemiDoc XRS+ Imaging System (Bio-Rad, USA) and the Image Lab Software (Edition 5.2; Bio-Rad, USA). Band intensity was normalized to total protein on native membrane, therefore no loading controls were necessary.

### Tissue fixation and histological preparation

Hearts were fixed in 3.7% formalin (Thermo Fisher Scientific, USA) for 48 h immediately after explantation. Fixed organs were dehydrated with ethanol and xylene, embedded in paraffin and cut into 4 μm sections for subsequent procedures.

Hematoxylin and eosin (HE) staining was performed, using a staining kit (Morphisto, Germany), following manufacturer’s instructions.

For immunohistochemical (IHC) assessment, paraffin sections were deparaffinized in xylene and ethanol, boiled twice in sodium citrate buffer (pH 6.0) for epitope retrieval and treated with 10% goat serum (Jackson Laboratories, USA) for 30 min for blockade of unspecific epitopes. Afterwards, the samples were washed in tris-buffered saline (TBS) and then incubated for 1 h at room temperature with the following antibodies (S1 Table): Anti-Cx43, anti-HMGB1, anti-cleaved-Caspase-3 (all Cell Signaling Technologies, USA). For detection of primary antibodies, alkaline-phosphatase conjugated goat anti-rabbit antibody (Jackson Laboratories, USA) was used and antibody conjugates were visualized with a red chromogen (Dako REAL Detection System, Alkaline Phosphatase/RED; Agilent Technologies, USA). The stained sections then were dehydrated in ethanol and xylene, mounted with NeoMount (Merck Millipore, Germany) and glass covered for long-term preservation.

To control for unspecific staining, sections were prepared with unspecific Immunoglobulin G and replacing primary antibodies, secondary antibodies and chromogen with TBS, respectively.

### Immunoflourescence preparation

For immunoflourescence, paraffin was removed from the sections, which were rehydrated prior to epitope retrieval procedure, as described above. Afterwards, the sections were treated with 10% goat serum for 2 h to prevent unspecific binding of antibodies. Specimens were then exposed to primary anti-Cx43 antibody (Cell Signaling Technologies, USA) for 60 min at room temperature and subsequently to Alexa Flour 568 labeled goat anti-rabbit secondary antibody (Thermo Fisher Scientific, USA) for 60 min.

Sections were mounted with ProLong Gold Antifade/ 4',6-diamidino-2-phenylindole (DAPI; Thermo Fisher Scientific, USA) for counterstaining of nuclei and long-term preservation.

### Histopathology and IHC analysis

To evaluate morphological damage and to rule out direct cardiac damage through air-blast injury and cardiac contusion, whole transversal HE sections were investigated for: 1) apoptosis, 2) contraction band necrosis, 3) neutrophilic infiltration, 4) intramuscular bleeding, 5) rupture, 6) edema and 7) ischemia (S2 Table).

To quantify epitope expression, images of five distinct, representative fields of view (100x magnification) were examined for each animal using an Imager.M1 microscope, EC Plan-NEOFLUAR objectives and an AxioCam MRc camera (all Zeiss, Germany) and the colorimetric density of the red signal was measured using the Zeiss Axio Vision software (Edition 4.9; Zeiss, Germany). For comparison of groups, mean values were calculated for each animal. Results are presented as densitometric sum red (DSR; arbitrary units). Positive and negative controls were used for calibration of measurement.

### Statistics

Statistical testing was performed using Sigma Plot software (Edition 11.0; Systat Software, Germany). Data sets were analysed for distribution pattern and equality of variance prior to testing. In cases of parametrical distribution, statistical significance was evaluated using the unpaired t-test for comparison of differences between group means and results are presented as group means ± standard error of the mean (SEM). In cases of non-parametrical distribution or inequality of variances, Mann-Whitney rank sum test was performed. These results are presented as median and quartiles. Values of p<0.05 were considered statistically significant.

## Results

### Early increase in systemic mediators of inflammation after murine polytrauma and hemorrhagic shock (PTHS)

**Fig 1:**
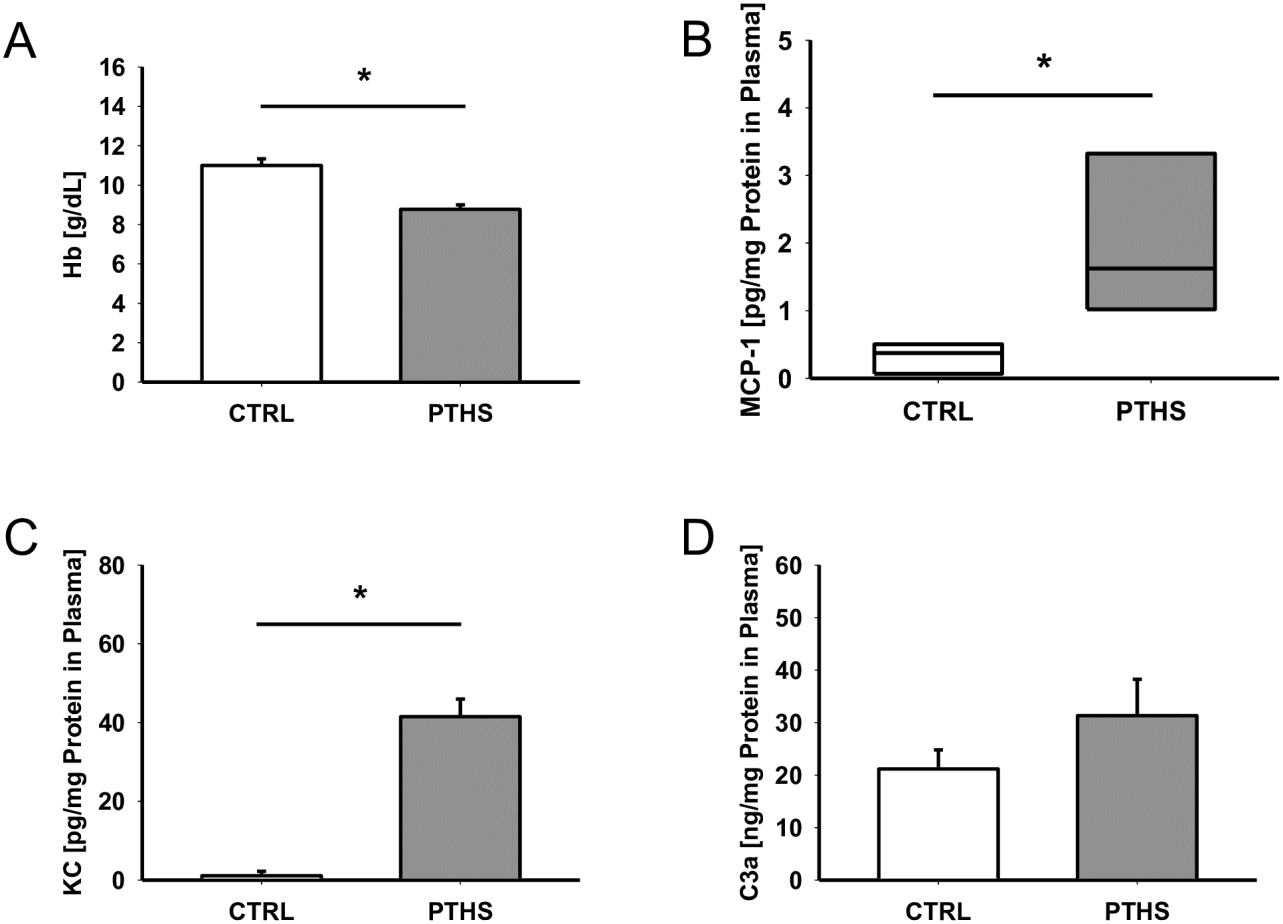
Plasma levels of inflammatory mediators and hemoglobin. Levels of (A) haemoglobin (Hb) in blood and (B) macrophage chemotactic protein 1 (MCP-1), (C) keratinocyte chemoattractant (KC) and (D) complement component C3a in plasma of animals 4 h after infliction of polytrauma and hemorrhagic shock (PTHS; n = 7 for Hb, n = 8 for MCP-1, KC and C3a) and native control animals (CTRL; n = 8 for Hb, n = 4 for MCP-1, n = 5 for C3a and KC). Results (B, C, D) are presented as amount of protein per total protein in plasma to unmask diluting effects from volume resuscitation. For statistical comparison of experimental means, unpaired t-tests (A, C, D) and Mann-Whitney rank sum test (B) were performed. *: p<0.05.

To assess the impact of PT, HS and volume resuscitation, the hemoglobin levels were determined and expectedly revealed significantly lower levels 4 h after PTHS (Fig 1A). Plasma concentrations of the pro-inflammatory cytokine MCP-1 (Fig 1B) and chemokine KC (Fig 1C) were significantly increased in PTHS animals 4 h after hemodynamically instable multiple trauma in comparison to CTRL. The central complement activation product C3a was slightly but non-significantly enhanced as early as 4 h after the traumatic insult (Fig 1D).

### Early signs of cardiac subcellular damage after PTHS

**Fig 2:**
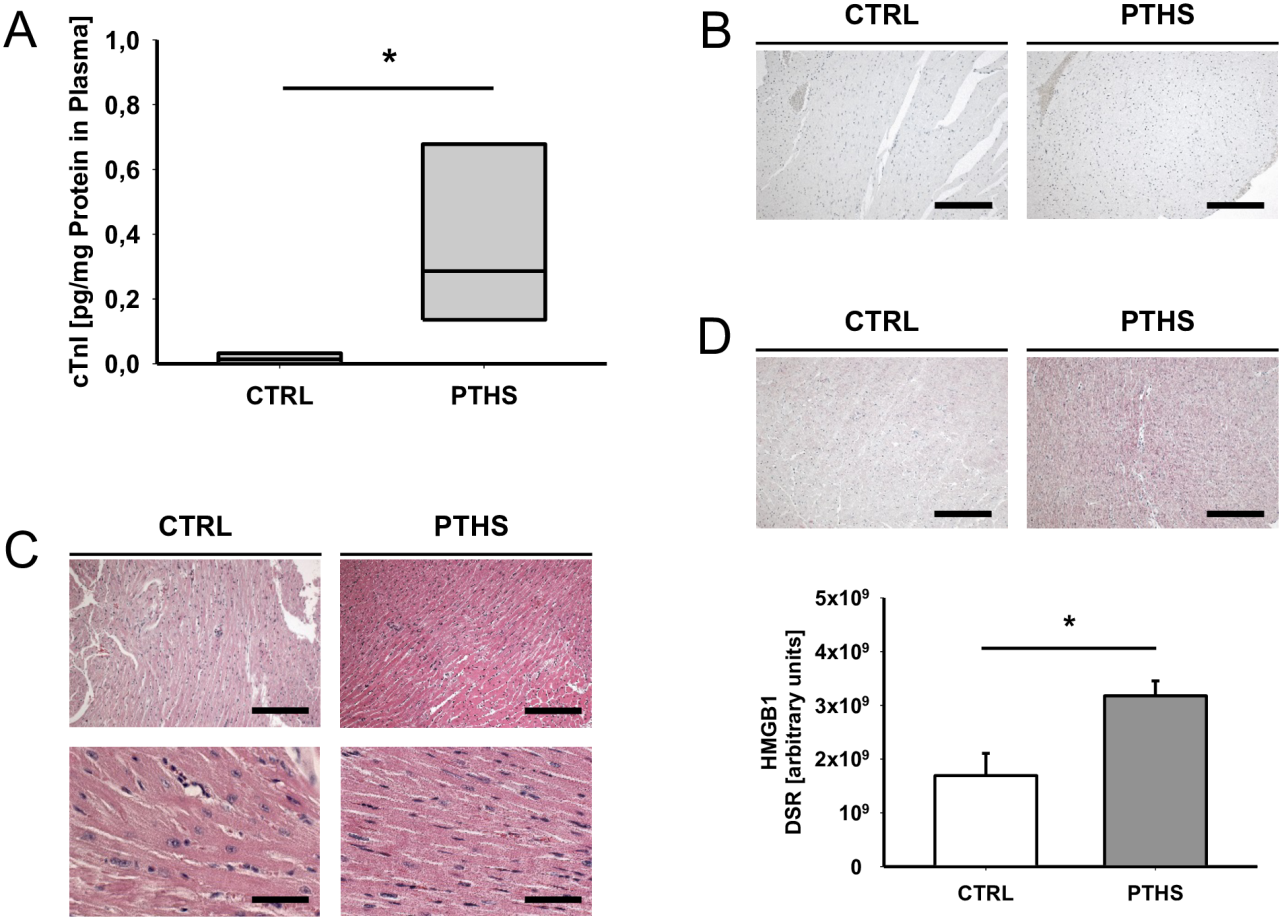
Histological and humoral markers of cardiac damage. (A) Plasma levels of cardiac troponin I (cTnI) as a marker of cardiac damage from animals 4 h after the insult of polytrauma and hemorrhagic shock (PTHS; n = 8) and native controls (CTRL, n = 4). Results are presented as amount of protein per total protein in plasma to unmask diluting effects from volume resuscitation. (B) Representative images of immunohistochemical (IHC) assessment of tissue cleaved caspase 3. Magnification: 100x (bar: 50 μm). (C) Representative hematoxylin and eosin stained sections of cardiac tissue, showing no signs of marked histomorphological changes. Magnifications: 100x (upper images, bar: 50 μm) and 200x (lower images, bar: 100 μm) for each group. (D) Representative images and densitometric analysis of IHC preparations of tissue high-mobility group box 1 (HMGB1) showing increased signal in samples from PTHS animals. Magnification: 100x (bar: 50 μm). For histological evaluation: n = 5 (PTHS); n = 5 (CTRL). DSR: density sum red. Experimental means were compared for statistical significance using the unpaired t-test (D) and Mann-Whitney rank sum test (A). *: p<0.05.

Plasma cTnI levels in plasma were significantly increased early after experimental PTHS (Fig 2A). However, histomorphological analysis of HE section did not show any significant signs of bleeding, cellular damage or necrosis, nor of leukocyte infiltration (Fig 2C, S3 Table). In line, IHC assessment of apoptosis by detection of cleaved caspase 3 could rule out induction of apoptosis in any group (Fig 2B). In contrast, IHC analysis of the nuclear protein HMGB1, which serves as a sensitive marker for cell damage, revealed a significant increase in the heart tissue of the PTHS group compared to controls (Fig 2D). Together, these findings indicate subcellular damage by PTHS that cannot be detected by standard histomorphological assessment.

### Altered intercellular distribution of the gap junction protein Cx43 after PTHS

**Fig 3:**
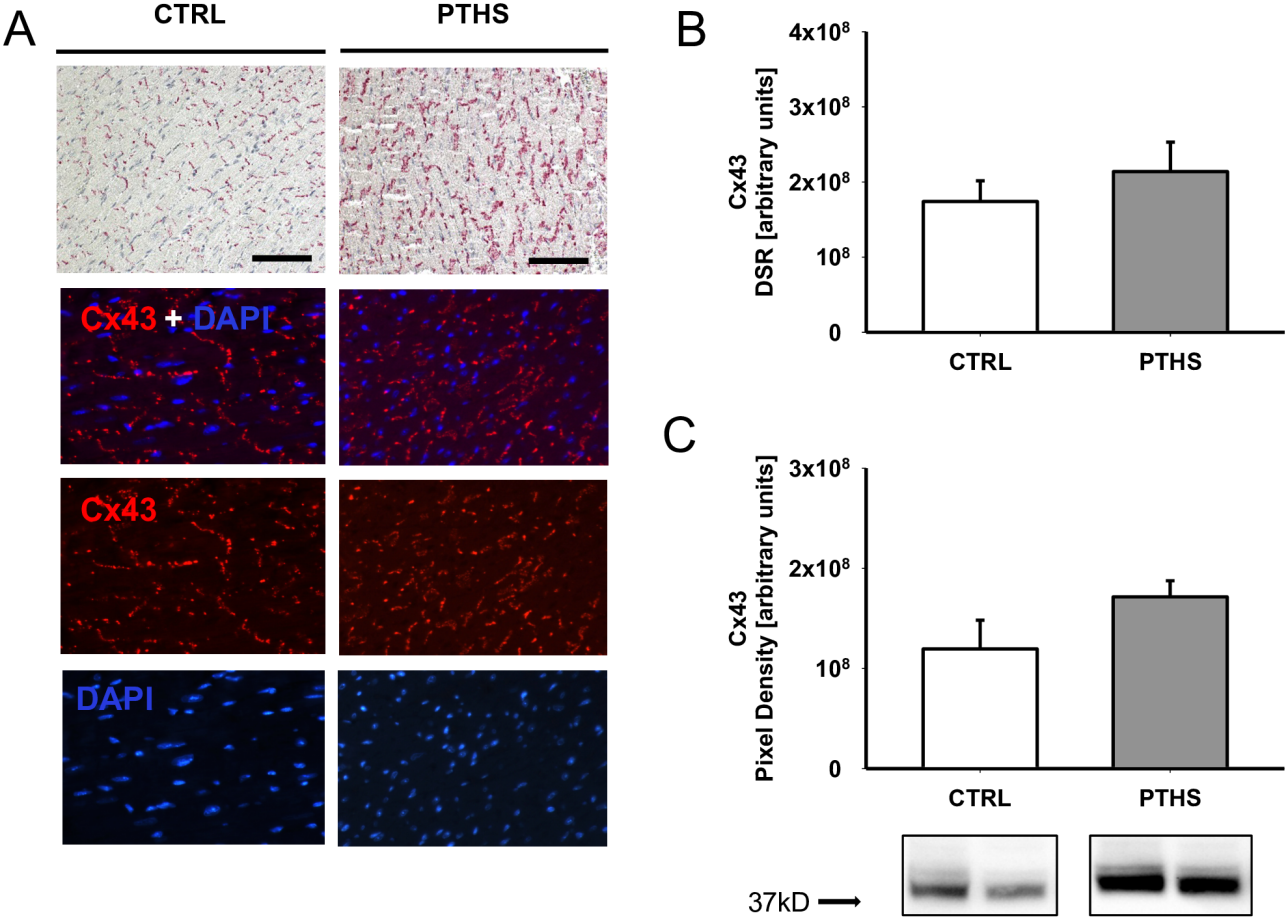
Morphological alterations of cardiac gap junction proteins. (A) Representative images of immunohistochemical (IHC) and immunofluorescence (IF) assessment of connexin 43 (Cx43) in cardiomyocytes show marked lateralization and disruption of gap junctions 4 h after experimental polytrauma and hemorrhagic shock (PTHS, n = 6) compared to native controls (CTRL, n = 5). Magnification (IHC): 100x (bar: 50 μm). Magnification (IF): 200x. (B) Densitometric analysis of IHC red signal and (C) evaluation of total amount of Cx43 in tissue homogenates by western blotting show slightly increased mean values for PTHS. Insets show representative bands from the same blot for CTRL and PTHS. n = 6 (PTHS); n = 4 (CTRL). DSR: density sum red. Experimental means were compared by unpaired t-test. *: p<0.05.

Animals of the PTHS group displayed marked areas of gap junction disintegration in longitudinal histological sections. These findings were consistent throughout all trauma animals and were scarcely observed in control mice (Fig 3A). Densitometric analysis of the IHC staining signal of Cx43 did not show significant differences between the experimental groups (Fig 3B). Detection of Cx43 proteins by western blotting revealed slightly increased band intensities for the PTHS group samples compared to CTRL (Fig 3C).

## Discussion

To the best of our knowledge, the present study is the first to present data on the effects of systemic inflammation in mice as early as 4 h after combined PT and HS (PTHS) on the cellular structures of cardiomyocytes and their intercellular junctions.

Significant increases in plasma cTnI levels in the present study suggest marked injury of cardiomyocytes caused by PT and HS. Elevated cTnI plasma levels have also been described in sepsis and septic shock [14,15] and are associated with the development of myocardial dysfunction and negative outcome [16]. It was proposed, though never proven, that the release of cTnI in conditions such as systemic inflammation may not be due to the loss of cardiac cells but to immediate, stress-triggered release of soluble cTnI from the myocardial cytoplasm [17]. In the present PT setting, there was certainly a significant systemic inflammatory response as reflected by the enhanced cytokine profile (Fig 1) and the appearance of DAMPs, including HMGB-1, as shown recently by our group [13].

However, in the present study, no morphological signs of direct cardiac damage were visible in HE standard preparations. Furthermore, all samples were negative for IHC evaluation of cleaved caspase 3 ruling out major early apoptotic events. On the other hand, there was a significant increase in heart tissue HMGB1 after PT and HS compared to controls, implying an early local and/or remote stress-response of the cardiac myocytes to trauma and subsequent systemic inflammation. HMGB1 is elevated in plasma of humans early after trauma and may drive systemic inflammation [18]. In an ischemia/reperfusion injury model, HMGB1 expression was increased in mouse hearts and associated with cardiomyocyte apoptosis [19]. Mechanistically, HMGB1 is also known to impair myocyte contractile function by reducing intracellular Ca^2+^-flow and by draining the intra-sarcoplasmatic Ca^2+^-pool [10]. Because crucial autosecretory effects have been described for the pro-inflammatory agents like interleukin 6 in septic hearts [20] it is tempting to speculate similar effects exist for HMGB-1 on cardiac cells. However, based on current literature and the present results, we cannot be sure whether the measured tissue-residual HMGB1 was precipitated from plasma-derived HMGB1 and/ or directly generated from cardiac cells, and whether it plays a crucial role in trauma patients’ cardiac function or for their overall outcome.

Detrimental effects of systemic inflammation on the CGJ and particularly on Cx43 have been established for years. Cx43 misdistribution has been held responsible for occurring cardiac depression and arrhythmias, especially after sepsis [21]. Cx43 lateralization along the cell membrane of cardiomyocytes has been described in septic patients with multi-organ failure [22]. The impact of sterile systemic inflammatory processes early after multiple injuries on the integrity of the CGJ has not been attended to until recently. In the present study, CGJ disruption showed as early as 4 h after trauma. We were also able to detect a slight although non-significant increase of total Cx43 protein in heart homogenates, suggesting an initiation of upregulation of Cx43 expression following injury and inflammation. It remains to be clarified whether in the later course after PTHS there is a more pronounced effect on Cx43 expression. Reduction of gap junction protein levels in sepsis and ischemia/reperfusion injury, [23,24] and down-regulation of Cx43 mRNA expression in rat hearts early after lipopolysaccharide injection [21] have been described. The somewhat contradictory findings described above suggest a different role of Cx43 early after trauma and sterile inflammation. As cardioprotective effects of Cx43 have been described in vivo [25] and in vitro [26], particularly in the context of ischemia, the slight increase in protein levels of Cx43 may be a sign of an early counter regulation in response to cardiomyocyte damage. It may also be triggered indirectly through circulating inflammatory mediators conditioning the heart prior to expected systemic stress following trauma. Also, upregulation of Cx43 occurred after administration of beta-adrenergic receptor agonists [27], a further hint to an endogenous response to acute stress.

In addition to determining quantitative alterations, it may be beneficial to conduct further studies to assess chemical changes of the Cx43 protein and their impact on CGJ function.

### Limitations of the study

Because of the low number of animals per group, the power of the study is below the necessary power for statistical testing. Therefore, p-values should be interpreted with caution and be considered exploratory, not confirmatory. Furthermore, there is a lack of functional data on cardiac activity such as echocardiography. Therefore, although the results of the present study may represent structural changes, to what extent these translate to cardiac dysfunction must be addressed in future studies.

## Conclusion

The present study demonstrates molecularly manifesting injury of cardiac muscle cells in the early phase after PT and HS in the setting of systemic inflammatory response. In addition, marked disruption of the CGJ was observed, associated with beginning changes in total Cx43 protein expression. Spatial and quantitative alteration of this important component of the electrical conduction system of the heart may lead to electrophysiological impairment and consequently to arrhythmia and cardiac dysfunction. Awareness of potentially developing cardiomyopathy after polytrauma may improve clinical handling of admitted patients and ameliorate outcome. However, further studies are still needed to widen our knowledge on the pathophysiological backgrounds and potential therapies.

## Supporting information

S1 Table Antibodies used for protocols. Cat.-No: catalogue number of manufacturer; HMGB1: high mobility group box 1; pc: polyclonal; mc: monoclonal; Asp: aspartic acid; Ala: alanine; IHC: immunohistochemistry; WB: western blotting; IF: immunofluorescence.
(DOCX)

S2 Table Damage scoring of hematoxylin and eosin stained sections. (DOCX)

S3 Table Histomorphological evaluation. Median score (1. quartile; 3. quartile) for the left (LV) and right (RV) ventricle of each group (CTRL: n = 5; PTHS: n = 5). For each animal, the whole tissue specimen was analyzed and scored. Data was analyzed for normality using the Shapiro-Wilk test and differences in medians were analyzed using one-way analysis of variance on ranks. CBN: contraction band necrosis. NS: Not statistically significant.
(DOCX)

## References

[1] Mokdad AH, Forouzanfar MH, Daoud F, Mokdad AA, El Bcheraoui C, Moradi-Lakeh M, et al Global burden of diseases, injuries, and risk factors for young people's health during 1990–2013: a systematic analysis for the Global Burden of Disease Study 2013. Lancet. 2016;387: 2383–2401. doi: 10.1016/S0140-6736(16)00648-6 27174305 10.1016/S0140-6736(16)00648-6

[2] Murray CJ, Lopez AD. Alternative projections of mortality and disability by cause 1990–2020: Global Burden of Disease Study. Lancet. 1997;349: 1498–1504. doi: 10.1016/S0140-6736(96)07492-2 9167458 10.1016/S0140-6736(96)07492-2

[3] Wen Y, Yang H, Wei W, Shan-shou L. The outcomes of 1120 severe multiple trauma patients with hemorrhagic shock in an emergency department: a retrospective study. BMC emergency medicine. 2013;13 Suppl 1: S6 doi: 10.1186/1471-227X-13-S1-S6 23902600 10.1186/1471-227X-13-S1-S6 PMC3701514

[4] Lenz A, Franklin GA, Cheadle WG. Systemic inflammation after trauma. Injury. 2007;38: 1336–1345. doi: 10.1016/j.injury.2007.10.003 18048040 10.1016/j.injury.2007.10.003

[5] Hoesel LM, Niederbichler AD, Ward PA. Complement-related molecular events in sepsis leading to heart failure. Molecular Immunology. 2007;44: 95–102. doi: 10.1016/j.molimm.2006.06.009 16875736 10.1016/j.molimm.2006.06.009

[6] Celes MRN, Prado CM, Rossi MA. Sepsis: Going to the Heart of the Matter. Pathobiology. 2012;80: 70 doi: 10.1159/000341640 22986917 10.1159/000341640

[7] Gawande N, Tumram N, Dongre A. Cardiac Changes in Hospitalized Patients of Trauma. Shock. 2014;42: 211–217. doi: 10.1097/SHK.0000000000000194 24827391 10.1097/SHK.0000000000000194

[8] Kalbitz M, Pressmar J, Stecher J, Weber B, Weiss M, Schwarz S, et al The Role of Troponin in Blunt Cardiac Injury After Multiple Trauma in Humans. World Journal of Surgery. 2016 doi: 10.1007/s00268-016-3650-7 27501709 10.1007/s00268-016-3650-7

[9] Asavarut P, Zhao H, Gu J, Ma D. The role of HMGB1 in inflammation-mediated organ injury. Acta Anaesthesiol Taiwan. 2013;51: 28–33. doi: 10.1016/j.aat.2013.03.007 23711603 10.1016/j.aat.2013.03.007

[10] Zhang C, Mo M, Ding W, Liu W, Yan D, Deng J, et al High-mobility group box 1 (HMGB1) impaired cardiac excitation-contraction coupling by enhancing the sarcoplasmic reticulum (SR) Ca(2+) leak through TLR4-ROS signaling in cardiomyocytes. J Mol Cell Cardiol. 2014;74: 260–273. doi: 10.1016/j.yjmcc.2014.06.003 24937603 10.1016/j.yjmcc.2014.06.003

[11] McCain ML, Desplantez T, Geisse NA, Rothen-Rutishauser B, Oberer H, Parker KK, et al Cell-to-cell coupling in engineered pairs of rat ventricular cardiomyocytes: relation between Cx43 immunofluorescence and intercellular electrical conductance. Am J Physiol Heart Circ Physiol. 2012;302: 443. 10.1152/ajpheart.01218.2010 PMC3339855 22081700

[12] Tao L, Liu HR, Gao F, Qu Y, Christopher TA, Lopez BL, et al Mechanical traumatic injury without circulatory shock causes cardiomyocyte apoptosis: role of reactive nitrogen and reactive oxygen species. Am J Physiol Heart Circ Physiol. 2005;288: 2811. 10.1152/ajpheart.01252.2004 15695560

[13] Denk S, Weckbach S, Eisele P, Braun CK, Wiegner R, Ohmann JJ, et al Role of Hemorrhagic Shock in Experimental Polytrauma. Shock. 2017 doi: 10.1097/SHK.0000000000000925 28614141 10.1097/SHK.0000000000000925

[14] ver Elst KM, Spapen HD, Nguyen DN, Garbar C, Huyghens LP, Gorus FK. Cardiac troponins I and T are biological markers of left ventricular dysfunction in septic shock. Clin Chem. 2000;46: 650–657. 10794747

[15] Altmann DR, Korte W, Maeder MT, Fehr T, Haager P, Rickli H, et al Elevated cardiac troponin I in sepsis and septic shock: no evidence for thrombus associated myocardial necrosis. PLoS One. 2010;5: e9017 doi: 10.1371/journal.pone.0009017 20140242 10.1371/journal.pone.0009017 PMC2815772

[16] Mehta NJ, Khan IA, Gupta V, Jani K, Gowda RM, Smith PR. Cardiac troponin I predicts myocardial dysfunction and adverse outcome in septic shock. Int J Cardiol. 2004;95: 13–17. doi: 10.1016/j.ijcard.2003.02.005 15159032 10.1016/j.ijcard.2003.02.005

[17] Korff S, Katus HA, Giannitsis E. Differential diagnosis of elevated troponins. Heart. 2006;92: 987–993. doi: 10.1136/hrt.2005.071282 16775113 10.1136/hrt.2005.071282 PMC1860726

[18] Cohen MJ, Brohi K, Calfee CS, Rahn P, Chesebro BB, Christiaans SC, et al Early release of high mobility group box nuclear protein 1 after severe trauma in humans: role of injury severity and tissue hypoperfusion. Crit Care. 2009;13: R174 doi: 10.1186/cc8152 19887013 10.1186/cc8152 PMC2811903

[19] Xu H, Yao Y, Su Z, Yang Y, Kao R, Martin CM, et al Endogenous HMGB1 contributes to ischemia-reperfusion-induced myocardial apoptosis by potentiating the effect of TNF-alpha/JNK. Am J Physiol Heart Circ Physiol. 2011;300: 913. 10.1152/ajpheart.00703.2010 PMC3302194 21186276

[20] Atefi G, Zetoune FS, Herron TJ, Jalife J, Bosmann M, Al-Aref R, et al Complement dependency of cardiomyocyte release of mediators during sepsis. FASEB J. 2011;25: 2500–2508. doi: 10.1096/fj.11-183236 21478262 10.1096/fj.11-183236 PMC3114524

[21] Fernandez-Cobo M, Gingalewski C, Drujan D, De Maio A. Downregulation of connexin 43 gene expression in rat heart during inflammation. The role of tumour necrosis factor. Cytokine. 1999;11: 216–224. doi: 10.1006/cyto.1998.0422 10209069 10.1006/cyto.1998.0422

[22] Takasu O, Gaut JP, Watanabe E, To K, Fagley RE, Sato B, et al Mechanisms of cardiac and renal dysfunction in patients dying of sepsis. Am J Respir Crit Care Med. 2013;187: 509–517. doi: 10.1164/rccm.201211-1983OC 23348975 10.1164/rccm.201211-1983OC PMC3733408

[23] Celes MR, Torres-Duenas D, Alves-Filho JC, Duarte DB, Cunha FQ, Rossi MA. Reduction of gap and adherens junction proteins and intercalated disc structural remodeling in the hearts of mice submitted to severe cecal ligation and puncture sepsis. Crit Care Med. 2007;35: 2176–2185. 17855834 10.1097/01.ccm.0000281454.97901.01

[24] Martins-Marques T, Catarino S, Zuzarte M, Marques C, Matafome P, Pereira P, et al Ischaemia-induced autophagy leads to degradation of gap junction protein connexin43 in cardiomyocytes. Biochem J. 2015;467: 231–245. doi: 10.1042/BJ20141370 25605500 10.1042/BJ20141370

[25] Bian B, Yu X, Wang Q, Teng T, Nie J. Atorvastatin protects myocardium against ischemia-reperfusion arrhythmia by increasing Connexin 43 expression: A rat model. Eur J Pharmacol. 2015;768: 13–20. doi: 10.1016/j.ejphar.2015.09.023 26386290 10.1016/j.ejphar.2015.09.023

[26] Yasui K, Kada K, Hojo M, Lee JK, Kamiya K, Toyama J, et al Cell-to-cell interaction prevents cell death in cultured neonatal rat ventricular myocytes. Cardiovasc Res. 2000;48: 68–76. 11033109 10.1016/s0008-6363(00)00145-0

[27] Xia Y, Gong KZ, Xu M, Zhang YY, Guo JH, Song Y, et al Regulation of gap-junction protein connexin 43 by beta-adrenergic receptor stimulation in rat cardiomyocytes. Acta Pharmacol Sin. 2009;30: 928–934. doi: 10.1038/aps.2009.92 19574999 10.1038/aps.2009.92 PMC4006640

