# Supplementary material for: Early structural changes of the heart after experimental polytrauma and hemorrhagic shock
Source: PLoS One. 2017 Oct 30;12(10):e0187327. doi: 10.1371/journal.pone.0187327 (PMC5662170; doi:10.1371/journal.pone.0187327)
Supplement: S1 Table — Cat.-No: catalogue number of manufacturer; HMGB1: high mobility group box 1; pc: polyclonal; mc: monoclonal; Asp: aspartic acid; Ala: alanine; IHC: immunohistochemistry; WB: western blotting; IF: immunofluorescence. (DOCX) [file pone.0187327.s001.docx]

| **Target** | **Product/ Cat.-No** | **Origin** | **Clonality** | **Source Antigen** | **Protocol/ Dilution** |
| --- | --- | --- | --- | --- | --- |
| Connexin 43 | Connexin 43 Antibody; Cell Signaling Technologies; #3512S | rabbit | pc | human Connexin 43 | IHC/ IF (1:100); WB (1:1000) |
| Cleaved Caspase 3 | Cleaved Caspase 3 (Asp175) Antibody; Cell Signaling Technologies; #9661 | rabbit | pc | synthetic peptide (amino-terminal residues adjacent to Asp175 in human caspase-3) | IHC (1:100) |
| HMGB1 | HMGB1 (D3E5); Cell Signaling Technologies; #6893 | rabbit | mc | synthetic peptide (residues surrounding Ala137 of human HMGB1 protein) | IHC (1:200) |

**S1 Table. Antibodies used for protocols.** Cat.-No: catalogue number of manufacturer; HMGB1: high mobility group box 1; pc: polyclonal; mc: monoclonal; Asp: aspartic acid; Ala: alanine; IHC: immunohistochemistry; WB: westernblotting; IF: immunofluorescence.
