# Supplementary material for: Early structural changes of the heart after experimental polytrauma and hemorrhagic shock
Source: PLoS One. 2017 Oct 30;12(10):e0187327. doi: 10.1371/journal.pone.0187327 (PMC5662170; doi:10.1371/journal.pone.0187327)
Supplement: S2 Table — (DOCX) [file pone.0187327.s002.docx]

| **Item** | **Score** |
| --- | --- |
| **Apoptosis** | 0, not present, single cell events  1, groups of cells, unifocal  2, groups of cells, several foci  3, wide spread apoptosis |
| **Contraction band necrosis** | 0, not present  1, single focus  2, multiple foci |
| **Neutrophilic infiltration** | 0, not present  1, single focus  2, multiple foci  3, wide spread infiltration areas |
| **Intramuscular bleeding** | 0, not present  1, single focus, occasional lesions  2, multiple lesions  3, extensive rupture/ bleeding, destruction of myocardium |
| **Rupture** | 0, not present  1, small areas of detachment  2, spread areas of detachment  3, extensive rupture bleeding, destruction of myocardium |
| **Edema** | 0, not present  1, single focus, occasional lesions  2, multiple foci |
| **Ischemia** | 0, not present  1, single focus, occasional lesion  2, multiple lesions |

**S2 Table. Damage scoring of hematoxylin and eosin stained sections.**
