# Supplementary material for: Early structural changes of the heart after experimental polytrauma and hemorrhagic shock
Source: PLoS One. 2017 Oct 30;12(10):e0187327. doi: 10.1371/journal.pone.0187327 (PMC5662170; doi:10.1371/journal.pone.0187327)
Supplement: S3 Table — Median score (1. quartile; 3. quartile) for the left (LV) and right (RV) ventricle of each group (CTRL: n = 5; PTHS: n = 5). For each animal, the whole tissue specimen was analyzed and scored. Data was analyzed for normality using the Shapiro-Wilk test and differences in medians were analyzed using one-way analysis of variance on ranks. CBN: contraction band necrosis. NS: Not statistically significant. (DOCX) [file pone.0187327.s003.docx]

**S3 Table. Histomorphological evaluation.** Median score (1. quartile; 3. quartile) for the left (LV) and right (RV) ventricle of each group (CTRL: n=5; PTHS: n=5). For each animal, the whole tissue specimen was analyzed and scored. Data was analyzed for normality using the Shapiro-Wilk test and differences in medians were analyzed using one-way analysis of variance on ranks. CBN: contraction band necrosis. NS: Not statistically significant.

|  | **CTRL** |  | **PTHS** |  | **p-Value** |
| --- | --- | --- | --- | --- | --- |
|  | **LV** | **RV** | **LV** | **RV** |  |
| Apoptosis | 1 (0.5;1) | 0 (0;1) | 0.5 (0;1.75) | 0.5 (0;1) | NS |
| CBN | 0 (0;0) | 0 (0;0) | 0 (0;0) | 0 (0;0) | NS |
| Infiltration | 0 (0;0) | 0 (0;0) | 0 (0;0) | 0 (0;0) | NS |
| Bleeding | 0 (0;0) | 0 (0;0) | 0.5 (0;1) | 0 (0;0) | NS |
| Rupture | 0 (0;0) | 0 (0;0) | 0 (0;0) | 0 (0;0) | NS |
| Edema | 1 (0.5;1) | 0 (0;0.5) | 1.5 (0.25;2) | 0.5(0;1) | NS |
| Eosinophilisation | 1 (0.5;1) | 0 (0;1) | 1.5 (1;2) | 1 (0.25;1) | NS |
